# Understanding serine and glycine metabolism in cancer: a path towards precision medicine to improve patient’s outcomes

**DOI:** 10.1007/s12672-024-01544-6

**Published:** 2024-11-13

**Authors:** Anaís Sánchez-Castillo, Kim R. Kampen

**Affiliations:** 1 Department of Radiation Oncology (MAASTRO), GROW School for Oncology and Reproduction, Maastricht University Medical Center, Maastricht University, Maastricht, The Netherlands; 2 Department of Oncology, Laboratory for Disease Mechanisms in Cancer, KU Leuven and Leuven Cancer Institute (LKI), Louvain, Belgium

## Abstract

In this perspective, we highlight and reflect on the current knowledge with respect to serine/glycine metabolism in cancer, therapeutic resistance, and precision medicine opportunities for therapeutic targeting and treatment follow-up. Cancer subtypes with high mortality rates include lung cancer and glioblastomas. In order to improve future therapeutic opportunities, patient stratification need to be performed to select patients that might benefit from adjuvant serine/glycine targeting compounds. In an effort to identify the group of patients for stratification purposes, we analyzed publicly available TCGA patient datasets to test associations between serine/glycine metabolism enzyme expression and important cancer drivers in lung cancer and glioblastoma. These patients presenting serine/glycine pathway overexpression might benefit from adjuvant sertraline treatment in the future.

## Cancer complexity

Cancer is characterized by the uncontrolled proliferation of cells, driven by genomic defects in regulatory mechanisms that control normal cell growth and homeostasis. As these cells progress towards a neoplastic state, they acquire a series of cancerous hallmarks. Originally, these hallmarks were defined as the essential capabilities of cancer cells that enable tumor growth and metastatic dissemination and ultimately, the development of malignant tumors. These encompassed the acquired capabilities of cancer cells for sustaining proliferative signaling, evading growth suppressors, resisting cell death, enabling replicative immortality, supporting angiogenesis, and activating invasion and metastasis [1]. The increasing knowledge of tumor biology led to the introduction of reprogramming cellular metabolism and avoiding immune destruction to the growing list of emerging cancer hallmarks. The authors also described the term “enabling characteristics”, representing means by which cancer cells and tumors can acquire and sustain these functional traits. Among these enabling processes were genome instability and tumor-promoting inflammation [2]. In addition, new potential hallmarks including unlocking phenotypic plasticity, non-mutational epigenetic reprogramming, polymorphic microbiomes, and senescent cells, were recently reviewed [3]. Our understanding of tumor biology now extends beyond the traits of cancer cells alone and requires the inclusion of the tumor microenvironment and its significant contribution to the process of tumorigenesis [4]. Interestingly, beyond focusing on the local tumor microenvironment, there is a growing recognition of the importance of the systemic microenvironment and the interplay of cancer cells and the immune system, which adds another layer of complexity and heterogeneity to cancer research [5].

Early detection and personalized treatment regimens are key factors in minimizing the burden and progression of cancer [6–8]. Conventional treatment approaches include surgery, radiotherapy, and chemotherapy, as single treatments or in combination. Radiotherapy and genotoxic chemotherapeutic agents, induce reactive oxygen species (ROS) and DNA strand breaks-mediated cell death, thereby enhancing the requirements of cancer cells for nucleotides and mechanisms to overcome ROS-mediated oxidative stress [9–14]. Yet, besides causing cancer heterogeneity, their impact on normal cells causes therapy side effects and toxicity [15–17]. To overcome therapeutic resistance it is essential to consider that cancer heterogeneity and plasticity result in the development of resistance mechanisms promoted by the selective pressure upon treatments [18, 19]. In the case of chemo- and radiation therapies, cancer cells, located at nutrient-deprived and oxygen-restricted tumor areas, have evolved to replenish their nucleotide pools and resist oxidative stress by adapting their metabolic pathways and coping with the therapy-induced stressors [14, 20–22]. As such, an often-undetectable cancer subclone will become resistant to these conventional treatment modalities, creating selective pressure, eventually allowing this subpopulation of cells to progress and metastasize.

## High mortality; therapeutic failure

Consequently, despite significant advances in understanding the genetic drivers of cancer and the improvements made in diagnosis and treatments with multimodal regimens, some types of cancer still present poor prognosis and high mortality rates, e.g., non-small cell lung cancer (NSCLC) and glioblastoma multiforme (GBM). These cancers are characterized by generally poor responses to current conventional, targeted treatments, and immune therapies, presenting high rates of adverse effects and the development of resistance mechanisms to cancer treatments. To overcome these obstacles, investigating resistance mechanisms and exploring multimodality therapies that can synergistically attack the tumor from several directions, including targeting these metabolic resistance mechanisms, represent major areas of focus in cancer research.

Lung cancer is the leading cause of cancer-related deaths worldwide, and NSCLC accounts for approximately 85% of lung cancer cases. The current standard of care for patients with NSCLC includes surgical resection, radiotherapy, chemotherapy, targeted therapies, and immunotherapy [23, 24]. NSCLC treatment options evolved over the last decade, presenting improved outcomes based on the concept of precision medicine. In the metastatic setting, targeted therapies have emerged targeting oncogenic drivers such as KRAS^G12C^, EGFR, ALK, ROS1, BRAF^V600^, MET, RET, and NTRK. More recently, standard-of-care treatment for patients lacking first-line targeted therapy options has included immunotherapy, using the immune checkpoint inhibitor anti-PD-(L)1 [25]. While targeted therapy and immunotherapy have led to significant improvements in outcomes within several months to years, collectively, more than 50% of patients with NSCLC still die of lung cancer within 5 years [23].

GBM represents one of the greatest challenges in the treatment of cancer patients worldwide, remaining deadly even with aggressive treatment schedules, including tumor resection, followed by radiotherapy and chemotherapy using temozolomide [26]. The development of treatments in GBM has focused on targeting molecular alterations commonly found in GBM [27, 28]. For example, therapeutic approaches have been aimed at targeting EGFR amplifications, which is the most common genetic alteration in GBM, occurring in approximately 50% of the cases, as well as its variant EGFRvIII, a mutation found in a subset of GBM cases [29]. Unfortunately, attempts to suppress EGFR activity by tyrosine kinase inhibitors, targeting EGFRvIII by vaccination using a peptide vaccine or antibody–drug conjugates have failed to yield positive clinical outcomes [30–32]. Apart from targeted therapies, immunotherapy approaches were unsuccessful in improving the outcome of GBM [33]. Phase III clinical trials using PD-1 inhibitor nivolumab did not show any benefit in overall survival in recurrent and newly diagnosed GBM [34–36]. Altogether, the median survival of patients with GBM remains only approximately 15 months.

## Metabolic reprogramming of cancer cells

**Fig. 1:**
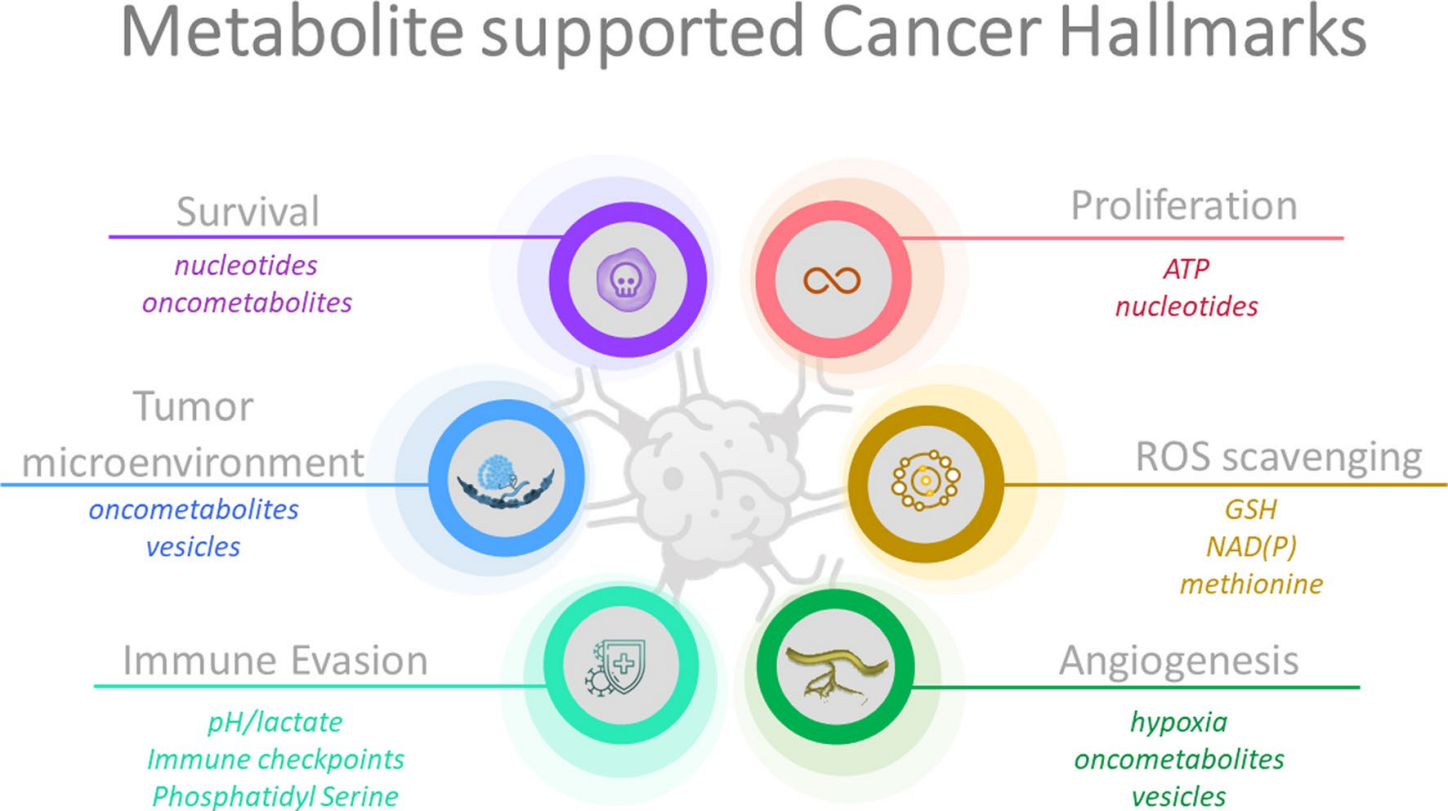
Cancer hallmarks that are regulated and linked to cancer cell metabolic reprogramming

Metabolic reprogramming is one of the hallmarks of cancer, with a crucial role in the initiation, proliferation, progression, and survival of cancer cells (Fig. 1). Among the molecular factors that mediate metabolic reprogramming of cancer cells are oncogenes, e.g., KRAS, MYC, NKX2-1 [37–39], loss-of-function of tumor suppressors, e.g., TP53, PTEN, STK11/LKB1 [40–44], but also hypoxia-induced factors (HIFs), along with signaling pathways and transcriptional networks, e.g. PI3K/mTOR pathway [45–49]. Cancer cells are selective in their import of nutrients from their environment and reprogram their metabolism to produce energy, maintain a reduction–oxidation (redox) balance, and generate the metabolites that they require for their continuous proliferation. At the same time, the unlimited proliferation of cancer cells leads to the depletion of nutrients and oxygen from their microenvironment since the high demand for nutrients and oxygen surpasses the resources in the microenvironment. Consequently, cancer cells need to adapt and become dependent on metabolic pathways that allow them to overcome the extreme conditions in the tumor microenvironment, including limited nutrient supply, low oxygen availability (hypoxia), and acidic pH Cancer cells also display higher production of cellular reactive oxygen species (ROS) compared to normal cells, in part due to increased metabolic rates and hypoxia. Regulation of ROS homeostasis is essential to control cell proliferation, survival, and differentiation, and a moderate increase in ROS contributes to tumor progression. However, high levels of ROS are capable of triggering cancer cell death. Consequently, cancer cells adapt their metabolism to upregulate the pathways that support the production of antioxidants, such as NADPH and glutathione (GSH), limiting the damaging effects of ROS, and thereby supporting the survival and proliferation of cancer cells and subsequent resistance to standard-of-care treatments [50–53].

**Fig. 2:**
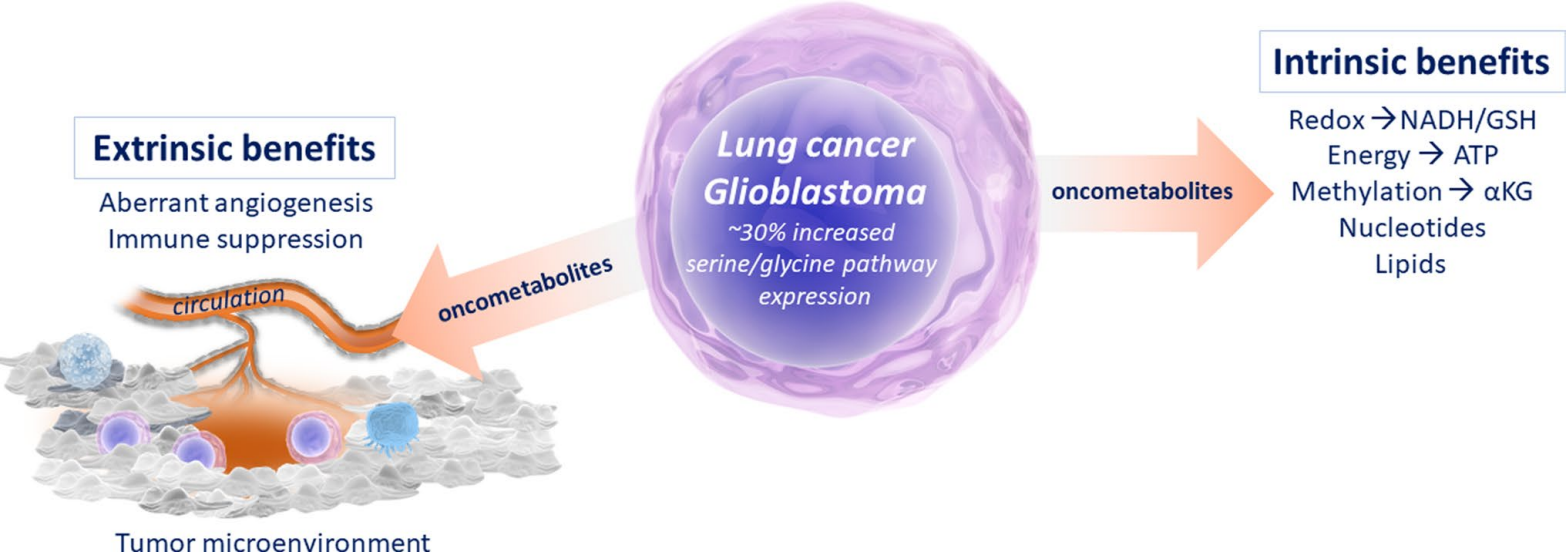
Cancer cell metabolism intrinsic benefits and influences on the tumor microenvironment

During disease progression, the accessibility of cancer cells to nutrients and oxygen is challenged, attributed to the reduced proximity to the vasculature and the formation of chaotic and non-structured vascularized networks due to aberrant tumor angiogenesis [54–56]. Additionally, the tumor-associated altered vasculature induces dynamic fluctuations in blood flow, and consequently, oxygen availability in distinctive diffuse patterns, a phenomenon known as cycling hypoxia [57, 58]. Consequently, the combination of these factors leads to the limitation of oxygen and nutrients in the tumor microenvironment in a dynamic and heterogeneous manner. Cancer cells are capable of metabolically adapting to these stress conditions, conferring them and their environment with a selective survival advantage (Fig. 2). The strategies that cancer cells employ consist in adopting alternative metabolic pathways and resources to obtain nutrients, and include; (1) increased endocytosis-based uptake of extracellular proteins and lipids and their subsequent intracellular degradation [59–62]; (2) symbiotic metabolic interactions between tumor cells with distinct metabolic requirements by exporting metabolites into the microenvironment and initiating cross-feeding mechanisms [63–65]; (3) enhanced autophagy, which involves lysosome-mediated degradation of nonessential intracellular components as a recycling source of amino acids, nucleotides and lipids [66–71]; (4) elevated (immune)proteasome function for protein degradation [72]; and (5) improved ribosome specialized function for selective translation, such as internal ribosome entry site (IRES)-mediated translation in response to cellular oxidative stress conditions [73, 74].

## De novo serine/glycine synthesis metabolic reprogramming

Besides elevated glycolysis, the glycolytic-side branching serine/glycine synthesis pathway is overexpressed in approximately 30% of all cancers (Fig. 2). Serine and glycine are two non-essential amino acids that can be synthesized via the de novo serine/glycine synthesis pathway and/or obtained from the microenvironment. In de novo serine synthesis, the glycolytic intermediate 3-phosphoglycerate is converted into serine via three consecutive enzymatic reactions, catalyzed by phosphoglycerate dehydrogenase (PHGDH), phosphoserine aminotransferase 1 (PSAT1) and phosphoserine phosphatase (PSPH). After de novo serine synthesis, the reversible interconversion of serine to glycine is catalyzed by either the cytosolic or mitochondrial serine hydroxymethyltransferase (SHMT), SHMT1 or SHMT2, respectively. In turn, the conversion of serine to glycine provides carbon units for one-carbon metabolism. Serine/glycine synthesis and downstream one-carbon metabolism pathways are involved in the synthesis of proteins, lipids, nucleic acids, methylation reactions, and maintenance of redox systems [75]. In a subset of cancers, genomic alterations in the genes encoding enzymes of the serine/glycine synthesis pathway result in the overexpression of these enzymes [75–77]. This leads to an increased influx of glycolytic intermediates into the de novo serine/glycine synthesis pathway. Apart from direct genomic alterations in serine and glycine synthesis genes, oncogenes and tumor-suppressor genes, e.g., KRAS^G12D^, MYC, NKX2-1, STK11 (LKB1), and NRF2-ATF4, have been shown to regulate the expression of the enzymes of the serine/glycine synthesis pathway [39, 78–81]. In contrast, normal cells and tissues have lower demands for serine and glycine and largely lack expression and activity of the de novo synthesis pathway, relying on the uptake of serine and glycine from their microenvironment.

## Putative serine/glycine pathway driver genes in NSCLC and GBM

There is a strong need for precision medicine based novel stratification methods and adjuvant metabolic targeting treatments to overcome therapeutic resistance, especially in cancer with high mortality rates. The upregulation of de novo serine and glycine synthesis has been observed in cancers, such as NSCLC and GBM [81–84], associated with poor prognosis of these cancer types. As such, we explored the connection between driver genes of GBM and lung adenocarcinoma (LUAD), the most common type of NSCLC, and their expression of serine/glycine synthesis enzymes. For this, we used public genomic data of LUAD and GBM patients from The Cancer Genomic Atlas (TCGA) through cBioPortal. This analysis provides ample insights into the patients harboring distinct genetic alterations who exhibit upregulation of the serine/glycine metabolic pathway and may benefit from pharmacological inhibition of the serine/glycine pathway in combination with current standard-of-care treatments.

**Fig. 3:**
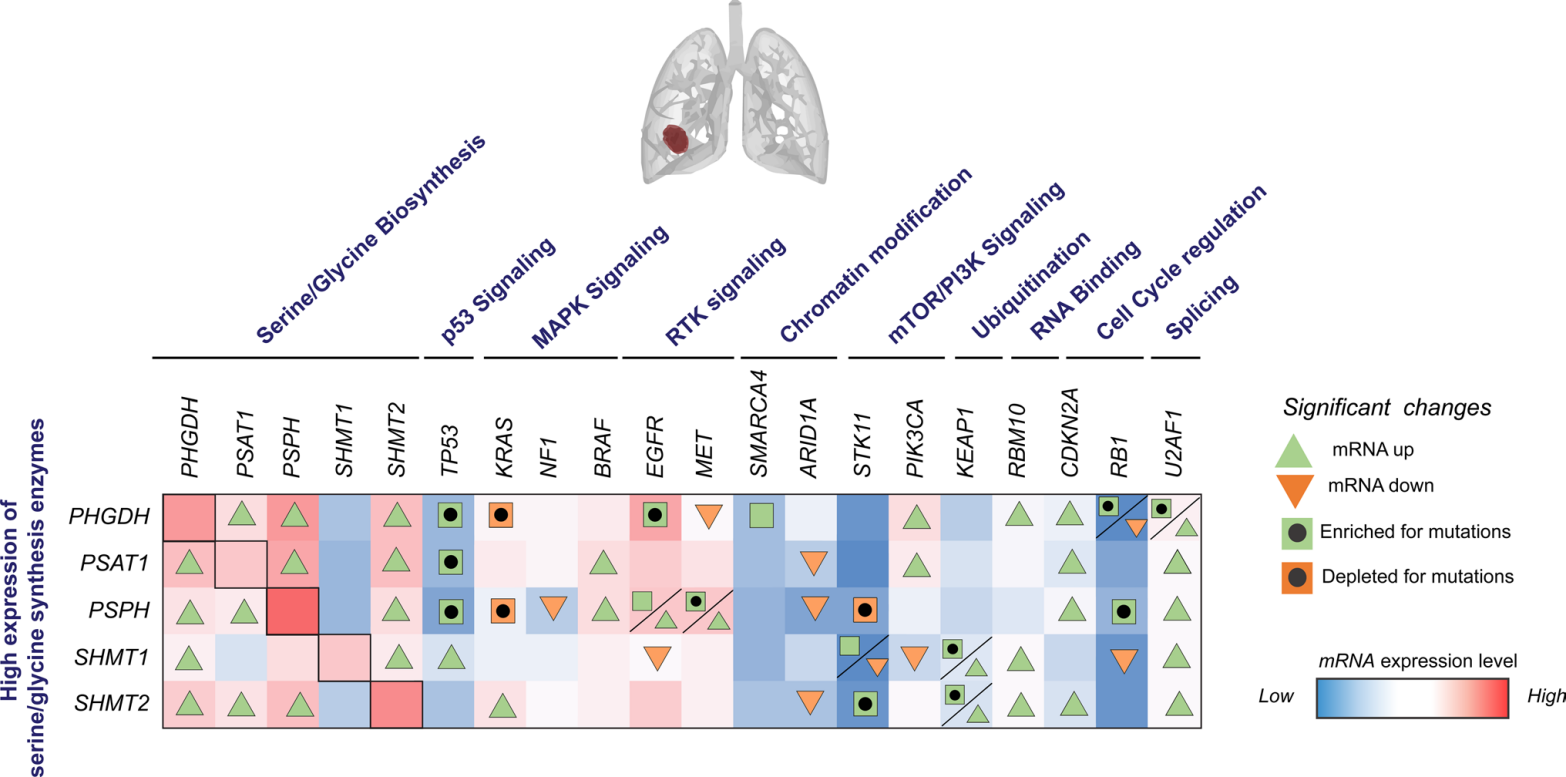
Link of serine/glycine synthesis enzyme overexpression with the expression and mutations of genetic drivers of LUAD. TCGA data of LUAD patients using TCGA, PanCancer Atlas dataset in cBioPortal, *n* = 503. The data was ranked according to high and low mRNA expression levels of serine/glycine synthesis enzymes, *PHGDH*, *PSAT1*, *PSPH*, *SHMT1,* and *SHMT2*, n = 150 high versus n = 150 low for each enzyme. The graph shows the sum of mRNA expression (high = red, low = blue) and mutation data of the main genetic drivers of LUAD in the patient groups with high expression of the different serine/glycine synthesis enzymes. The significant changes were calculated upon comparison of the mRNA expression and mutation data in the group of patients with high vs low expression of serine/glycine synthesis enzymes. Copy-number alterations (CNA) were not included due to a frequency lower than 10%. A two-tailed equal variance t-test has been performed for gene expression analysis and Fisher Exact test for mutations

EGFR is one of the most frequently mutated and/or amplified genes in both LUAD and GBM [85–87]. Activated EGFR leads to the induction of signaling cascades such as PI3K/AKT and RAS/RAF/MAPK pathways [88]. For example, EGFR mutant NSCLC cells upregulate glycolysis and pentose phosphate pathway (PPP) via the PI3K/AKT/mTOR signaling axis, which results in enhanced pyrimidine nucleotide synthesis and redox homeostasis [89, 90]. Serine synthesis dependency has also been reported as a metabolic vulnerability of EGFR-driven cancers, contributing to the synthesis of nucleotides and redox homeostasis. The upregulation of serine synthesis PHGDH and PSPH enzymes was observed in LUAD patients and patient-derived xenograft tumors harboring EGFR mutations. Moreover, the authors found co-occurrence of PSPH focal amplification and EGFR L858R mutation, which accounted for 16% of the EGFR mutant group. It is important to note that EGFR and PSPH are located in close chromosomal proximity at region Chr7p11.2. Additionally, only EGFR-driven cells were sensitive to the inhibition of serine synthesis using PHGDH inhibitors [91]. Interestingly, serine/glycine synthesis has been identified as a resistance mechanism to the first-generation EGFR tyrosine kinase inhibitor, erlotinib, in LUAD. Inhibition of PHGDH expression restored sensitivity to erlotinib in lung cancer cell lines and xenografts [92]. Consistent with these studies, we observed that the group of LUAD patients with high expression of *PHGDH* and *PSPH* presented an enrichment of mutated EGFR compared to the groups with low expression of these metabolic enzymes (Fig. 3). Moreover, the LUAD patient group with high expression of *PSPH* showed elevated expression of *EGFR*.

**Fig. 4:**
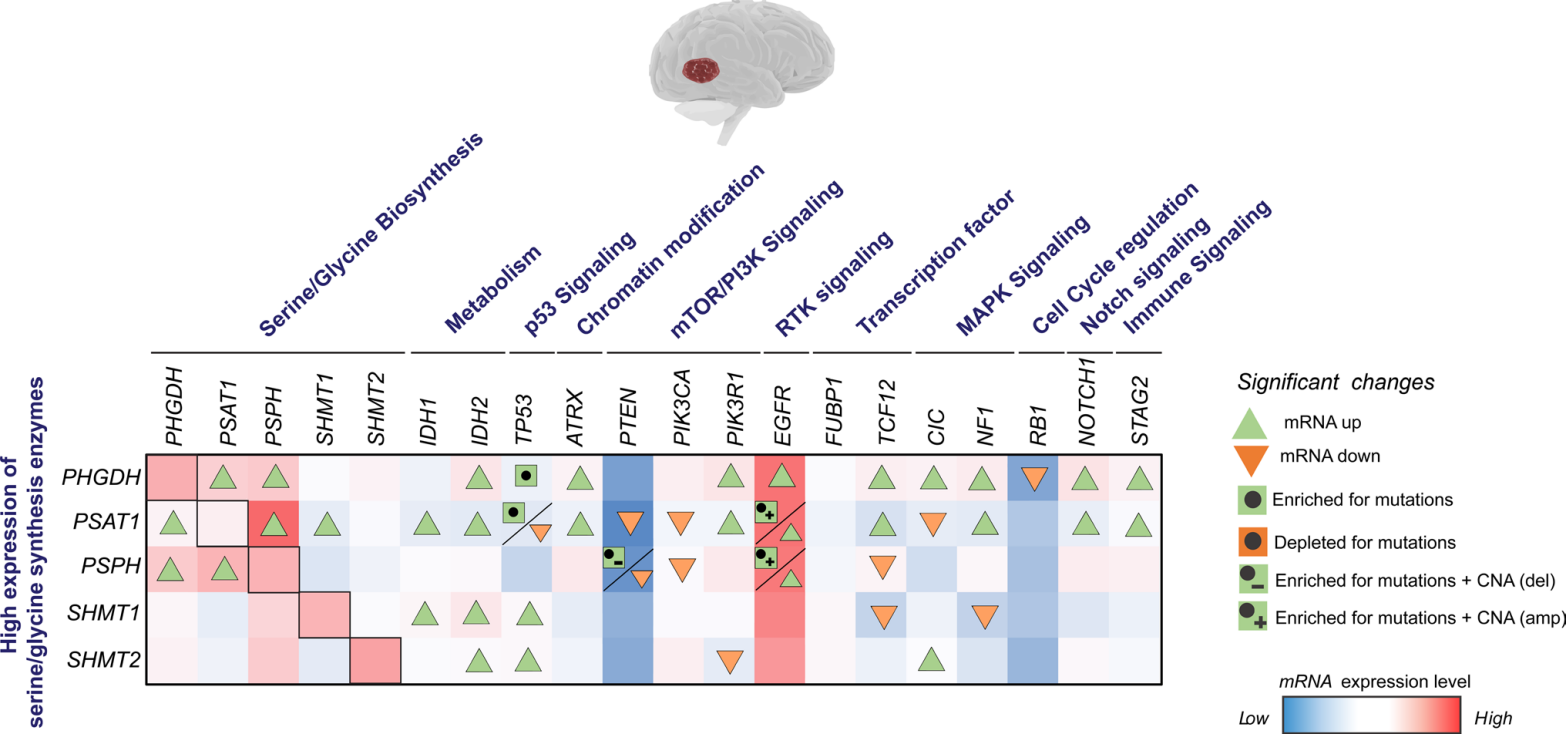
Link of serine/glycine synthesis enzyme overexpression and expression, mutations, and CNA of genetic drivers of GBM. TCGA data of GBM patients using TCGA, Firehose Legacy Samples with mRNA data (Agilent microarray) *n* = 401. The data was ranked according to high and low mRNA expression levels of serine/glycine synthesis enzymes, *PHGDH*, *PSAT1*, *PSPH*, *SHMT1,* and *SHMT2*, n = 150 high versus n = 150 low for each enzyme. Of note, among GBM patients with high expression of *PSPH*, 25% of these cases include PSPH amplifications. The graph shows the sum of mRNA expression (high = red, low = blue) and mutation/CNA data of the main genetic drivers of GBM in the patient groups with high expression of the different serine/glycine synthesis enzymes. The significant changes were calculated upon comparison of the mRNA expression and mutation/CNA data in the group of patients with high vs low expression of serine/glycine synthesis enzymes. CNA were included in those genes with a ≥ 10% frequency. A two-tailed equal variance t-test has been performed for gene expression analysis and Fisher Exact test for mutations/CNA

In the case of GBM, EGFR genomic alterations by mutations and amplifications have been shown to promote glycolysis, glutaminolysis, and lipid metabolism via MYC and mTOR [93–95]. However, EGFR-mediated activation of serine/glycine synthesis has not been reported. In our TCGA analysis, we observed that the GBM patient groups with high expression of *PHGDH*, *PSAT1,* and *PSPH* display higher expression of *EGFR* compared to those cases with lower expression of these serine/glycine metabolic enzymes (Fig. 4). In addition, high expression of *PSAT1* and *PSPH* patient groups are enriched for EGFR mutations and copy number amplifications.

The TP53 tumor suppressor gene has been a major focus in the context of cancer research, frequently mutated in cancer, including LUAD and GBM [96]. Moreover, given its role in the DNA damage repair pathway, p53 is strongly associated with resistance to radiotherapy [97–100]. The tumor suppressor p53 is involved in regulating different metabolic processes, including glycolysis, oxidative phosphorylation, glutaminolysis, and antioxidant responses [101–105]. In addition, p53 activation can promote cancer cell survival during metabolic stress conditions, such as nutrient deprivation or hypoxia. In lung cancer, the cytotoxic effect of the glycolytic inhibitor 2-deoxy-D-glucose (2DG) is enhanced in p53-defective cells, which are unable to produce ATP via oxidative phosphorylation and respond to oxidative stress via modulation of antioxidants when glycolysis is inhibited [106]. In GBM, the p53-induced glycolysis and apoptosis regulator (TIGAR) has been shown to provide protection to GBM cells in glucose and oxygen-restricted conditions via enhanced synthesis of antioxidants to overcome oxidative stress. The inhibition of TIGAR led to an increase in ROS levels and subsequent cell death under hypoxic conditions, as well as enhanced efficacy of irradiation and temozolomide treatments [107, 108]. Similarly, p53 has been implicated in the regulation of serine/glycine synthesis enzymes, as well as protection of cancer cells during serine starvation [109, 110]. Yet, the role of wildtype or mutant p53 in the regulation of serine/glycine synthesis in LUAD and GBM has not been investigated yet. We observed that LUAD patients with high expression of *PHGDH*, *PSAT1,* and *PSPH* show enrichment of mutated p53 compared to patients with low expression of these enzymes (Fig. 3). Similarly, the GBM patients with high expression of *PHGDH,* and *PSAT1* exhibited an enrichment of mutated p53. Additionally, the patient group with high expression of *PSAT1* showed lower expression of *p53* (Fig. 4).

RB1 is a tumor suppressor gene altered in LUAD and GBM and associated with tumor progression and poor prognosis [111–114]. Alternatively, RB1 protein can be inactivated by post-translational modifications, such as phosphorylation (pRB1) via the cyclin-dependent kinases CDK4/6 and CDK2 [115, 116]. Phosphorylated RB1 acts as a transcriptional co-repressor. In the nucleus, pRB1 binds and prevents DNA binding and transactivating functions of E2F1 [117]. These inhibitory functions can affect metabolic pathways such as glycolysis, glutaminolysis, and mitochondrial oxidative phosphorylation. In addition, pRB1 is involved in the synthesis of nucleotides and antioxidants [118–125]. The pRB1 has not been associated with serine/glycine synthesis in LUAD, nor GBM patients. In our analysis, we observed an enrichment of RB1 mutations in the group of LUAD patients with high expression of *PHGDH* and *PSPH*, as well as reduced expression of *RB1* (Fig. 3). In GBM, the patients with a high expression of *PHGDH* showed lower expression of *RB1* (Fig. 4).

Several genetic drivers of LUAD, i.e., KRAS, BRAF, KEAP1, and STK11, are linked to serine/glycine synthesis. NSCLC cells with KRAS^G12D/G12D^ mutations exhibit increased glycolysis and subsequently fuel glucose-derived metabolites into serine/glycine synthesis through the upregulation of nuclear factor erythroid-2–related factor 2 (NRF2). This was specifically observed in advanced tumor stages, providing these cells with enhanced NADP(H) and GSH-mediated antioxidant defenses [126]. NRF2 has been shown to transcriptionally activate *PHGDH*, *PSAT1,* and *SHMT2* genes via the activating transcription factor 4 (ATF4), a key mediator of stress responses, thereby supporting GSH and nucleotide production in NSCLC [81]. Interestingly, activated KRAS^G12D^ and BRAF^V619E^ NSCLC tumors induce *NRF2* gene transcription [127, 128]. Our TCGA analysis revealed elevated expression of *KRAS* in the LUAD patient group exhibiting high *SHMT2* expression. In contrast, we observed a depletion of KRAS mutations in the LUAD patient group with high expression of *PHGDH* and *PSPH* (Fig. 3), which may reflect mutation specificity for serine/glycine pathway upregulation or KRAS-linked serine-glycine conversion dependency. Additionally, groups with high expression of *PSAT1* and *PSPH* demonstrated elevated *BRAF* expression compared to patients with low expression of these serine/glycine synthesis genes. The tumor suppressor gene, *KEAP1,* is frequently mutated in LUAD and encodes an E3 ubiquitin ligase that negatively regulates the protein level of the NRF2 [85, 129]. Loss-of-function mutations in KEAP1 induce nuclear accumulation of NRF2, which has been associated with more aggressiveness and resistance to chemo- and radiation therapies in NSCLC [130, 131]. Interestingly, LUAD patients with high expression of *SHMT1* and *SHMT2* showed enrichment of KEAP1 mutations. Despite the expression levels of *KEAP1* being higher in these groups, mutations of KEAP1 lead to an inactive KEAP1 protein [132, 133], which may result in NRF2 nuclear accumulation and subsequent induction of serine/glycine synthesis in these cases (Fig. 3). Another tumor suppressor frequently mutated in LUAD is *STK11*, also known as *LKB1*, which encodes a kinase protein that regulates an important energy sensor in cancer cells, i.e., AMP-activated protein kinase (AMPK). The LKB1-AMPK pathway has been shown to promote cancer cell survival in response to acute nutrient stress by inhibiting mTOR, leading to the induction of autophagy [134, 135]. Furthermore, the simultaneous loss of LKB1 and KEAP1 boosts SHMT-driven antioxidant defence in KRAS-mutant lung cancer [44]. Our analysis revealed that within the LUAD patients exhibiting high expression levels of *SHMT1* and *SHMT2,* there was an increase in cases with mutations in STK11. Additionally, we observed lower expression of *STK11* in the group of patients with high *SHMT1* expression. However, LUAD patients with high expression of *PSPH* showed a decreased number of cases with mutations in STK11, suggesting a different regulatory mechanism in this case (Fig. 3).

In GBM, besides EGFR amplifications and mutations, IDH mutations and PTEN loss stand out as some of the most extensively investigated genetic drivers influencing the regulation of cancer cell metabolic reprogramming. IDH1 and IDH2 are metabolic enzymes that catalyze the reversible conversion of isocitrate to α-ketoglutarate (α-KG), using NADP as a cofactor to produce NADPH. Apart from being an intermediate of the TCA cycle, α-KG is required for the function of α-KG-dependent dioxygenases. Some examples of α-KG-dependent dioxygenases are histone lysine demethylases (KDMs), TET enzymes that mediate DNA demethylation, and prolyl hydroxylase domain (PHD) enzymes, regulators of HIFα. In GBM, neomorphic IDH1/2 mutations lead to the conversion of α-KG into the oncometabolite D-2-hydroxyglutarate (D-2-HG), which acts as an antagonist of α-KG, inhibiting the activity of α-KG-dependent dioxygenases [136, 137]. Therefore, mutations in IDH1/2 have been reported to alter DNA and histone methylation, as well as metabolic pathways, such as glycolysis, amino acid metabolism, lipid metabolism, and redox homeostasis [138–140]. In GBM, serine/glycine synthesis has not been associated with IDH. Our TCGA analysis of GBM patients revealed that the groups with elevated expression of *PHGDH*, *PSAT1*, *SHMT1,* and *SHMT2* exhibited higher expression levels of *IDH2*. Notably, only high *PSAT1* and *SHMT1* expression were associated with elevated levels of *IDH1* compared to patients with low *PSAT1* and *SHMT1* expression (Fig. 4).

Frequent PTEN mutations and deletions have also been reported in GBM [86, 141]. PTEN is a negative regulator of the PI3K signaling pathway by dephosphorylating the 3′ position of phosphatidylinositol-3,4,5-trisphosphate (PIP_3_) [142]. Loss of PTEN regulates tumor metabolism through the activation of PI3K/AKT/mTOR signaling axis, crucial for cell growth and survival [143]. PTEN has been shown to regulate glycolytic enzymes, promoting aerobic glycolysis in GBM [144]. In addition, PTEN mutations in GBM stem cells stimulate pyrimidine nucleotide synthesis by phosphorylation and activation of the enzyme CAD, mediated by the PI3K-AKT-mTOR-S6K pathway. The upregulation of pyrimidine synthesis in GBM stem cells correlated with tumor grade in patients with GBM. Targeting CAD enzymes results in the depletion of pyrimidine nucleotide supply, leading to the inhibition of stem cell survival, self-renewal, and in vivo tumor initiation [145]. Our TCGA analysis revealed that the GBM patient groups with high *PSAT1* and *PSPH* expression also present lower *PTEN* expression. Additionally, the patient group with high expression of *PSPH* shows enrichment for PTEN mutations and copy number deletions (Fig. 4).

Accumulating evidence has revealed serine/glycine synthesis metabolic reprogramming in multiple cancer subtypes, both intrinsically and upon exposure to harsh microenvironment conditions and cancer therapies. To fully exploit the therapeutic potential of inhibiting serine/glycine synthesis pathway, crucial requisites are patient stratification and a better understanding of the interplay between serine/glycine synthesis dependency in tumor cells and non-malignant cells within the tumor microenvironment. Patient stratification involves examining genomic alterations of serine/glycine synthesis enzymes as well as genetic drivers of enhanced serine/glycine synthesis pathway. Here, the association of expression of serine/glycine synthesis enzymes with multiple driver genes of LUAD and GBM highlights the significance of serine/glycine synthesis pathway activation in these tumor types as an advantageous mechanism that may contribute to tumor progression and resistance to treatments, as we previously observed in NSCLC [146].

## Future perspectives

Metabolomic analysis on patient’s tumor biopsies enables the exploration of metabolic reprogramming of cancer cells. However, technical limitations of metabolomic analyses, which often require the destruction of the tumor tissue, include the lack of a comprehensive characterization of tumor heterogeneity and the interplay with cells in the tumor microenvironment. New methodologies are being introduced to overcome this, such as spatial mass spectrometry imaging-based metabolomics, which allows for obtaining information about the content and spatial distribution of metabolites in tissues [147, 148]. Interestingly, this technology can be integrated with the exploration of spatially resolved multi-omics providing additional insights into the tumor microenvironment [149]. In addition, spatial metabolomics can be employed to discern the selectivity of metabolic-targeted compounds for tumor cells by examining the distribution of anticancer drugs in the tumor tissue, as well as to evaluate response to therapies [150].

The investigation of cellular metabolism often involves the utilization of stable isotopes, such as ^13^C-labeled metabolites or deuterated metabolites, e.g., glucose, glutamine, serine, or glycine, which offers information about the activity of metabolic pathways by following the integrative patterns of ^13^C and ^2^H enrichment in downstream metabolites [151]. Of note, these labeled-metabolite tracing strategies offer a safe approach to assess tumor metabolism in vivo in mice models and in patients; however, further expansion of their measurable metabolite repertoire is required for standard of care. For instance, intra-operative ^13^C-glucose infusions in NSCLC patients coupled with ^18^fluoro-2-deoxyglucose positron emission tomography (^18^FDG-PET) and multi-parametric MRI have been used to assess glucose fates and compare the metabolic requirements of cancer cells with those of the adjacent non-malignant lung cells [152]. ^18^FDG-PET is limited to glucose uptake, while the use of ^13^C-glucose allows us to obtain information about downstream metabolic pathways. These approaches provide valuable insights into the field of tumor metabolic reprogramming by depicting the metabolic requirements of cancer cells within the tumor microenvironment. Extensive efforts have been directed towards targeting enhanced glucose uptake and glycolysis in cancer cells [153–155]. Yet, the inhibition of this pathway has not been successfully included in clinical practice due to a limited therapeutic window related to normal tissue toxicity [156]. For example, the compound 2-deoxyglucose (2-DG) [157] has been investigated in clinical trials at lower doses aiming to reduce its toxicity. However, these doses proved insufficient to inhibit tumor progression [158, 159]. The use of ^18^FDG-PET imaging has revealed that the requirement of glycolysis and high glucose uptake is not a unique feature of tumors, with for instance the brain exhibiting high physiological FDG uptake [160, 161].

Metabolic-based anticancer therapies currently in use include chemotherapies known as antimetabolites, which are structural analogs of metabolites that interfere with the synthesis of nucleotides and DNA replication. The antifolates were the first class of antimetabolites, e.g., methotrexate and pemetrexed. These folate analogs inhibit folate-dependent reactions from one-carbon metabolism required for de novo nucleotide synthesis, thus disrupting DNA replication and inducing programmed cell death [162, 163]. The clinical efficacy of antifolates led to the development of purine and pyrimidine analogs [164, 165]. One example of these is 5-fluorouracil (5-FU), an analog of uracil that inhibits the enzyme thymidylate synthase, consequently impairing the synthesis of thymidine nucleotides and limiting its availability for DNA replication and repair [166]. These drugs have shown clinical success due to the increased demands of cancer cells for nucleotides for DNA replication. However, their lack of specificity for tumor cells leads to serious adverse side effects. Targeting cellular proliferation, these drugs affect many non-malignant cells such as those in bone marrow or gastrointestinal tract, which are rapidly proliferating cells. Consequently, myeloid suppression and gastrointestinal toxicity are observed as dose-limiting toxicities caused by antimetabolite chemotherapies [167]. Moreover, both intrinsic and acquired resistance to these therapeutic strategies presents an additional challenge. For instance, increased serine consumption and serine mitochondrial metabolism via SHMT2 enzyme have been shown to support purine biosynthesis and potentiate DNA damage repair in 5-FU-resistant colorectal cancer cells [168].

Given the crucial role of serine and glycine in the synthesis of nucleotides via one-carbon metabolism it is not surprising that efforts have been made to develop inhibitors of serine/glycine synthesis pathway. These serine/glycine synthesis enzyme inhibitors hold great potential as anticancer therapy because of the more specific mode of action than the clinically used antimetabolites that target one-carbon metabolism. PHGDH catalyzes the first step of de novo serine synthesis, which is amplified in some cancer types, such as melanoma, breast cancer, and NSCLC [75–77, 169]. Small-molecule inhibitors targeting PHGDH have been shown to inhibit serine synthesis and tumor proliferation in vitro and in xenograft cancer models [170–172]. However, inhibitors of PHGDH have a limited clinical perspective, as serine has an important role in the central nervous system and brain development and PHGDH deficiency can lead to neurological defects [173, 174]. Consequently, targeting SHMT and thereby the interconversion of serine to glycine might represent a more favorable strategy to avoid toxicity in the brain. The interconversion of serine to glycine by the enzyme SHMT1/2 donates one-carbon units to one-carbon metabolism [175–177]. Although inhibitors of SHMT1/2 have been developed, they present unfavorable pharmacokinetics, such as SHIN1. Alternatively, an improved derivative of SHIN1, known as SHIN2, has been predominantly investigated in T-cell acute lymphoblastic leukemia (T-ALL) [178–180]. Our group has repurposed the antidepressant sertraline as a dual SHMT1/2 inhibitor, showing both in vitro and in vivo efficacy on serine/glycine synthesis-dependent breast cancer, RPL10 R98S T-ALL and NKX2-1 T-ALL and NSCLC cell models [39, 73, 181]. The use of repurposed FDA-approved drugs, such as sertraline, presents promising opportunities as adjuvant anticancer therapeutic strategy since their safety and pharmacological profiles have been already established, which facilitates their translation into the clinic, as well as due to their cost-effectiveness. Serine/glycine synthesis-dependent cancers exhibit a greater sensitivity to the inhibition of this pathway using sertraline. These findings hold promise for minimizing normal tissue toxicity while maximizing therapeutic efficacy to be exploited in future combination treatments.

Analysis of metabolite plasma levels proves to be a promising strategy to monitor the response to therapies and to identify biomarkers of metabolic rewiring of cancer cells and therapy responses. For example, in high-grade gliomas, brain microdialysis fluids and serum during interstitial cisplatin treatment have been used to investigate the metabolic patterns associated with the treatment [182]. Metabolomic analysis on plasma samples of patients with stage I/III NSCLC defined a reduction of the antioxidant glutathione (GSH) during and after radiotherapy, which plays a key role in the protection of cancer cells from the oxidative stress induced by radiation therapy [146]. Serine/glycine-dependent cancers also systemically serve as a major resource for serine/glycine-derived metabolites. Notably, serine/glycine synthesis-dependent NKX2-1 tumor cells produce increased levels of triglycerides, which were also increasingly found in the blood of serine/glycine dependent RPL10 R98S mutant leukemia (T-ALL) patients. Furthermore, NKX2-1 tumor-bearing mice display increased blood levels of the antioxidant GSH [39]. Hence, serine, glycine, and serine/glycine synthesis-derived metabolites may serve as biomarkers for the diagnosis of serine/glycine synthesis-dependent cancers that consequently may benefit from the inhibition of serine/glycine synthesis using sertraline [26, 55, 163]. In addition, glycine metabolite levels in the tumor fluids could be used to predict the efficacy of sertraline in blocking serine to glycine conversion in cancer cells and thereby the subsequent export of glycine [146].

The implementation of these new technologies will allow us to better understand how cancer cells reprogram their metabolism towards de novo serine/glycine synthesis and its subsequent impact on metastasis, therapy resistance, and evasion from immune destruction. We propose that these cancers may benefit from the repurposed serine/glycine pathway inhibitor sertraline.

## Data Availability

Data used in this manuscript is publicly available and extracted from cBioportal. The respective datasets used for these analysis are anotated in the figure legends.
